# Enhancing learning skills in children by using assistive technology: a psychophysiological database

**DOI:** 10.1186/s13104-025-07499-3

**Published:** 2025-10-14

**Authors:** César E. Corona-González, Luz María Alonso-Valerdi, David I. Ibarra-Zarate, Fabiola R. Gómez-Velázquez

**Affiliations:** 1 Tecnologico de Monterrey, Escuela de Ingeniería y Ciencias, Ave. Eugenio Garza Sada, 2501, Sur, Col: Tecnológico, Monterrey, 64700 N.L México; 2 Instituto de Neurociencias, Universidad de Guadalajara, Francisco de Quevedo 180, Col. Arcos Vallarta, Guadalajara, 44130 Jalisco Mexico

**Keywords:** Assistive technology, EEG, Psychometrics, Learning difficulties, Smartick

## Abstract

**Objectives:**

The present dataset provides psychometric and electroencephalographic information from children with either reading or math difficulties. This data was collected before and after children utilized Smartick, an assistive technology that boosts reading and math proficiency. This dataset aims to understand how assistive technology can support learning in children.

**Data description:**

Seventy-six Mexican children aged 7 to 13 were recruited for this study. Every child underwent a psychometric assessment to identify learning difficulties. Then, each child was categorized within the group of reading difficulties or math difficulties. After that, children underwent electroencephalographic recording in two conditions: (1) resting state and (2) while performing reading or math activities. Each child was reallocated to (1) the experimental subgroup, which interacted with Smartick, and (2) the control subgroup, which did not enroll in the intervention. Finally, psychometric and electroencephalographic (EEG) data were collected again after a three-month follow-up period. All electroencephalographic recordings are presented in set format. The authors also share different .xlsx files describing: (1) psychometric results throughout the study, (2) the availability of electroencephalographic data, event list within the electroencephalographic recordings during (3) reading activities and (4) math activities, and (5) the engagement percentage with Smartick in the experimental group.

**Supplementary Information:**

The online version contains supplementary material available at 10.1186/s13104-025-07499-3.

.

## Objective

Learning difficulties are an increasingly frequent problem seen in classrooms. Despite the vast technological tools and pedagogical strategies to support learning, some children struggle with academic activities like reading and mathematics. Environmental factors such as socioeconomic status [1, 2], and family background [3] could magnify the severity of the problem. Consequently, children can face stress [4] and anxiety [5] for scholarly activities, low self-confidence [6], decreased motivation for learning [7], and school dropout [8]. Therefore, this study proposes the use of an assistive technology for learning called Smartick^1^ as a promising alternative to relieve these conditions. Before selecting a suitable learning technology, you should answer affirmatively the following questions: (a) Does it aim to develop academic skills? (b) Does it provide positive feedback? (c) Does the interface promote educational or ethical values? (d) Does it encourage active user engagement? (e) Is the technology gamified? (f) Is the interface user-friendly and aesthetic designed? (g) Is the technology age-appropriate [9]? . Smartick meets all these requirements, since (a) it includes training programs for reading and mathematics, (b) it provides positive feedback in response to user performance, (c) it promotes responsibility and commitment through daily use, (d) it requires active user interaction, (e) it features a fully gamified interface, (f) it has an aesthetic and user-friendly design and (g) it uses an adaptive learning approach.

Our methodology was designed to evaluate assistive technology’s impact on children’s learning skills. This dataset contains psychometric results and Electroencephalography (EEG) recordings. This data was collected before and after Smartick access was provided. Psychometric results could suggest improvements in learning performance, whereas EEG data can be useful to study the neurophysiological changes due to learning improvement, if any.

## Data description

Seventy-six children aged 7 to 13 years participated in this study. This work was developed in four stages. First, an initial psychometric evaluation was performed, where reading and math abilities were estimated. These assessments allowed each child to be enrolled in the Reading Difficulties Group (RDG) or Math Difficulties Group (MDG). Second, EEG recordings were collected under two states: (1) a 3-minute eyes-open resting-state condition and (2) while conducting either reading (for RDG) or math (for MDG) activities. Third, each group was subcategorized as the experimental group, where an intervention with Smartick was carried out, and the control group, which was not required to be involved with Smartick. Both groups were followed up for three months after the first EEG session. Finally, psychometric tests and EEG recordings were collected again.

EEG data was organized in a nested folder with three levels. The first level corresponds to the subject identifier. Each subject was labeled considering the main group, the subgroup, and a two-digits consecutive number. Uppercase letters R and M were used to indicate the main groups. Lowercase letters e and c denoted the subgroup (experimental and control, respectively. The second level splits data into the stage of the study in which the record was taken. EEG data from the initial psychophysiological assessment (PRE-intervention) were labeled as ses-1 while data from the final assessment (POST-intervention) were labeled as ses-2.

The third level describes the activity performed by the participant during EEG recordings. The resting-state condition is mentioned as run-1 for both reading and math difficulties groups. Additionally, we labeled the active reading and reading comprehension sections as run-2 and the presentation of the two blocks of arithmetic operations as run-2 and run-3. The following is an example of how the files were labeled.

- The file *sub-Rc03_ses-1_task-SmartickDataset_run-2_eeg.set* is related to the EEG recording from the third participant from the reading group and control subgroup during the PRE-intervention in the reading activity.
- Similarly, the participant *sub-Me02_ses-2_task-SmartickDataset_run-1_eeg.set* refers to the second participant from the math group and experimental subgroup evaluated during the POST-intervention in a resting state condition.

Furthermore, Excel files describe psychometric and EEG data information. The *01_Psychometric_Data.xlsx* file contains the psychometric results from the PRE- and POST-interventions; the *02_EEG_Data_Descriptor.xlsx* file shows the availability of EEG data; the *03_Reading_Tags.xlsx* and *04_Math_Tags.xlsx* files display the EEG event lists for the reading and math experimental paradigms, respectively; and the *05_SessionEngagement*.*xlsx* file exhibits how children in the experimental group were engaged within Smartick sessions during the intervention. Finally, the *32_Chs_Location.ced* file holds the channel coordinates.

EEG signals were recorded by two synchronized 16-channel USBAMP RESEARCH amplifiers. Moreover, EEG data was acquired through the OpenVibe [10] software (v3.3.1.) in .*gdf* format. Then, EEG signals were detrended and bandpass filtered (1–30 Hz, 8th Butterworth IIR) in EEGLAB [11]. No further preprocessing steps were applied. The authors suggest that this database could also be used to apply different EEG preprocessing strategies. EEG files were further stored in *set*-format (Table 1).

**Table 1:** Overview of data files/data sets

Label	Name of data file/data set	File types (file extension)	Data repository and identifier
Data file 1	01_Psychometric_Data	MS Excel file (.xlsx)	OpenNeuro (10.18112/openneuro.ds006260.v1.0.1) [12]
Data file 2	02_EEG_Data_Descriptor	MS Excel file (.xlsx)	OpenNeuro (10.18112/openneuro.ds006260.v1.0.1) [12]
Data file 3	03_Reading_Tags	MS Excel file (.xlsx)	OpenNeuro (10.18112/openneuro.ds006260.v1.0.1) [12]
Data file 4	04_Math_Tags	MS Excel file (.xlsx)	OpenNeuro (10.18112/openneuro.ds006260.v1.0.1) [12]
Data file 5	05_SessionEngagement	MS Excel file (.xlsx)	OpenNeuro (10.18112/openneuro.ds006260.v1.0.1) [12]
Data file 6	32_Chs_Location	Channel location file (.ced)	OpenNeuro (10.18112/openneuro.ds006260.v1.0.1) [12]
Data set 1	sub-Mc01 to sub-Mc22 folders *EEG from the MDG*,* control subgroup*	Dataset file (.set)	OpenNeuro (10.18112/openneuro.ds006260.v1.0.1) [12]
Data set 2	sub-Me01 to sub-Me19 folders *EEG from the MDG*,* experimental subgroup*	Dataset file (.set)	OpenNeuro (10.18112/openneuro.ds006260.v1.0.1) [12]
Data set 3	sub-Rc01 to sub-Rc16 folders *EEG from the RDG*,* control subgroup*	Dataset file (.set)	OpenNeuro (10.18112/openneuro.ds006260.v1.0.1) [12]
Data set 4	sub-Re01 to sub-Re19 folders *EEG from the RDG*,* experimental subgroup*	Dataset file (.set)	OpenNeuro (10.18112/openneuro.ds006260.v1.0.1) [12]
Data set 5	stimuli folder *(subfolder Math)*	Joint Photographic Experts Group file (.jpeg)	OpenNeuro (10.18112/openneuro.ds006260.v1.0.1) [12
Data set 6	stimuli folder *(subfolder Reading)*	Portable Network Graphics file (.png)	OpenNeuro (10.18112/openneuro.ds006260.v1.0.1) [12]

### Limitations

- The EEG events related to the reading comprehension questions are not embedded in the PRE-EEG files due to technical issues in the experimental design. However, the sequence for these questions was registered manually. This information is displayed in the “Question shown (PRE)” column in the *03_Reading_Tags.xlsx* file. The description of “Unavailable” can be found in this column, which means that the event was not registered at all.
- A total of 386 signals were recorded for this database. However, 41 EEG signals (10.62%) showed poor quality. The authors recommend not using these signals for formal analysis. This data is shared with the scientific community, for those who are willing to work with EEG preprocessing pipelines. Furthermore, 20 EEG signals (5.18%) were not recorded due to computational issues occurred when saving some EEG files. In the *02_EEG_Data_Descriptor.xlsx* file, the yellow cells indicate corrupted signals, while the red cells highlight missing signals.
- The recording site did not have an isolated ground, so the authors suggest adding an additional filter to smooth the signal and improve the signal-to-noise ratio (e.g., moving average filter).

## Supplementary Information

Below is the link to the electronic supplementary material.

Supplementary Material 1

## Data Availability

The data described in this Data note can be freely and openly accessed on OpenNEURO under the accession number ds006260. Please see Table 1 and reference [12] for details and links to the data.

## Footnotes

1
https://mx.smartickmethod.com/.

## Abbreviations

EEG: Electroencephalogram
MDG: Math Difficulties Group
RDG: Reading Difficulties Group
